# Endoscopic Enucleation versus Open Prostatectomy for Treating Large Benign Prostatic Hyperplasia: A Meta-Analysis of Randomized Controlled Trials

**DOI:** 10.1371/journal.pone.0121265

**Published:** 2015-03-31

**Authors:** Maoyin Li, Jianguang Qiu, Qi Hou, Dejuan Wang, Wentao Huang, Cheng Hu, Ke Li, Xin Gao

**Affiliations:** 1 Department of Urology, Third Affiliated Hospital of Sun Yat-Sen University, Guangzhou, China; 2 Department of Urology, Central Hospital of Longgang District, Shenzhen, China; Eberhard Karls University, GERMANY

## Abstract

**Objectives:**

To evaluate the overall efficacy and safety of endoscopic enucleation of the prostate (EP) vs open prostatectomy (OP) for large benign prostatic hyperplasia (BPH).

**Methods:**

We conducted an electronic search of PubMed/Medline, EMBASE, The Cochrane Library, and Web of Science to detect all relevant randomized controlled trials (RCTs) comparing EP with OP. A meta-analysis was performed using Review Manager 5.3.

**Results:**

Seven RCTs (735 patients) were included. At the 3-, 6- and 12-month follow-up, there were no significant differences in the International Prostate Symptom Score (IPSS), maximum flow rate (Qmax), quality of life (QoL) score and post-void residual urine volume (PVR) between EP and OP. The International Index of Erectile Function (IIEF-5) was higher with EP (weighted mean difference [WMD]: 1.00, 95% confidence interval [CI]: 0.21 to 1.78, p=0.01) at the 12-month follow-up. The catheterization time (WMD: 3.80 d, 95%CI: -5.11 to -2.48, P<0.00001) and hospital stay (WMD: 4.93 d, 95%CI: -5.96 to -3.89, P<0.00001) were shorter with EP. The duration of operation was longer for EP compared with OP (WMD: 16.21 min, 95%CI: 3.72 to 28.70, P=0.01). The resected tissue weight (WMD: -9.63 g, 95%CI: -14.46 to -4.81, P<0.0001) and decrease in hemoglobin (WMD: -1.14 g/dL, 95%CI: -1.81 to -0.47, P=0.0008) were less with EP. EP was associated with fewer blood transfusions (risk ratio: 0.22, 95%CI: 0.10 to 0.47, P=0.0001). There were no significant differences between EP and OP when comparing other complications.

**Conclusions:**

Although only a limited number of RCTs with relatively limited follow-up are available, EP is shown to have a similar postoperative profile and comparable safety to OP. By contrast, EP may have a more desirable perioperative profile. EP appears to be an effective and safe minimally invasive option for treating large prostates that requires only brief convalescence.

## Introduction

Benign prostatic hyperplasia (BPH) is one of the most common pathologic processes that contribute to lower urinary tract symptoms (LUTS) in elderly males [1]. LUTS may lower the quality of life and interfere with daily activities [2, 3]. Several autopsy studies have demonstrated that the prevalence of BPH rapidly increases at the age of 40, reaching a prevalence of nearly 100% at the age of 90 [4].

Surgery remains one of the most effective approaches for the management of BPH [5]. In two randomized controlled trials (RCTs), compared with the baseline values, open prostatectomy significantly reduced LUTS by 63–86%, improved the IPSS-QoL score by approximately 60–87%, increased the average Qmax by 375%, and reduced the post-void residual urine volume (PVR) by 86–98% [6, 7]. Transurethral resection of the prostate (TURP) has been the standard surgical therapy for LUTS suggestive of BPH for prostate sizes of 30–80 mL [5]. In cases involving markedly enlarged prostates (>80 mL), open prostatectomy (OP) is still considered to be the most effective and durable procedure available [5, 8]. However, OP is undoubtedly the most invasive approach and is associated with substantial intraoperative morbidity, which extends the catheterization time and length of hospital stay [8, 9].

In the past two decades, newer minimally invasive surgical treatment options for BPH have been developed [10]. Since holmium laser enucleation of the prostate (HoLEP) was first introduced in 1996 [11], HoLEP has become widely recognized as an effective and safe method for the treatment of large BPH [12]. Endoscopic enucleation is an increasingly popular option for the management of large BPH, and many contemporary lasers such as thulium [13] and diode [14] lasers have been used for enucleation. Currently, interest in bipolar electrosurgical enucleation of the prostate (BEEP) [15], which has met with a certain degree of initial success, has emerged in the medical field. The major advantage of endoscopic enucleation is the ability to remove the adenoma close to the anatomical plane between the surgical capsule and the adenoma for a gland of any size, similarly to what the index finger does during an OP procedure; additionally, the efficacy of this procedure is equivalent to that of OP [15, 16]. Furthermore, the real advantages of endoscopic enucleation are equivalent or even superior to OP. However, it remains to be determined whether endoscopic enucleation has the potential to replace OP as the first-line surgical treatment for large BPH.

Our objective is to conduct a quantitative meta-analysis of randomized controlled trials that compare endoscopic enucleation of the prostate (EP) with OP in large BPH. The prostate size in all trials is larger than 70 mL, and all open prostatectomies are transvesical approaches.

## Methods

### Literature search

A meta-analysis of the literature was conducted based on articles published between 1998 and 27 July 2014 on the management of BPH. A systematic search of electronic databases, including PubMed/Medline, EMBASE, The Cochrane Library and Web of Science, was performed on 27 July 2014 using the terms “prostatic hyperplasia OR prostate hypertrophy OR prostatic enlargement OR Urinary Bladder Neck Obstruction OR Prostate Adenoma OR benign prostatic hyperplasia OR bladder outlet obstruction” and “enucleation” and “open prostatectomy OR transvesical prostatectomy OR transvesical open prostatectomy OR transvesical open enucleation OR retropubic prostatectomy OR retropubic adenomectomy OR adenomectomy OR prostatectomy OR prostatectom*”. The search strategy was modified as required in each electronic database. Additionally, a full manual search of the references from relevant articles was also performed. Searches were not restricted by regions, publication status or language and included conference proceedings and abstracts.

### Study selection

The inclusion and exclusion criteria were defined beforehand. RCTs that met the following criteria were included: 1) evaluated the efficacy and safety of EP compared with OP; 2) included patients with symptomatic LUTS caused by BPH; 3) clearly documented clinical outcomes using tools such as urologic symptom scales or urodynamic measurements; and 4) included OP performed by a transvesical approach. The exclusion criteria were as follows: 1) included patients with neurogenic bladder, suspected prostate cancer or bladder tumors; 2) included patients with prostate volumes< 70 mL; 3) included patients with previous prostate or urethral surgery; and 4) lacked the data necessary to make calculations or estimations from the published results.

### Data extraction and methodological quality assessment

Studies were selected based on the pre-established inclusion criteria. Review of the identified abstracts was carried out by two independent authors. The full text was retrieved for any studies that appeared to meet the inclusion criteria. Independently, two reviewers used a standardized form to extract the following data: publication year; first author; comparator; trial size; follow-up; baseline characteristics, including age, prostate volume, serum prostate-specific antigen (PSA), International Prostate Symptom Score (IPSS), maximum flow rate (Qmax; mL/s), quality of life (QoL) score, post-void residual urine volume (PVR; mL) and International Index of Erectile Function (IIEF-5) before the operation; perioperative outcomes, including the operative time, specimen weight, hemoglobin drop, catheterization time and length of hospital stay; postoperative efficacious outcomes, including the Qmax, PVR, QoL and IPSS after surgery; and complications.

The methodological quality assessment of the included RCTs was based on the Jadad composite scale [17, 18]. Any discrepancies about trial eligibility and inclusion were resolved through discussion or arbitration involving an independent third reviewer.

### Statistical analysis

The weighted mean difference (WMD) and the risk ratio (RR) were used for continuous and binary outcomes, respectively. All data were reported with 95% confidence intervals (CIs). The overall effects were determined by the Z-test, and P<0.05 was used to define statistical significance. Statistical heterogeneity between studies was assessed by the Cochrane χ^2^-test and I^2^ statistics. If I^2^>50% or P<0.10 was detected, we considered the data to be heterogeneous, and a random effect model was used [19]. Otherwise, a fixed effect model was used [20]. The presence of publication bias was evaluated using funnel plots. We also conducted subgroup analyses to examine possible differences between each group. The statistical analysis was performed with Review Manager 5.3 (The Cochrane Collaboration, Oxford, United Kingdom).

## Results

### Description of studies

**Fig 1:**
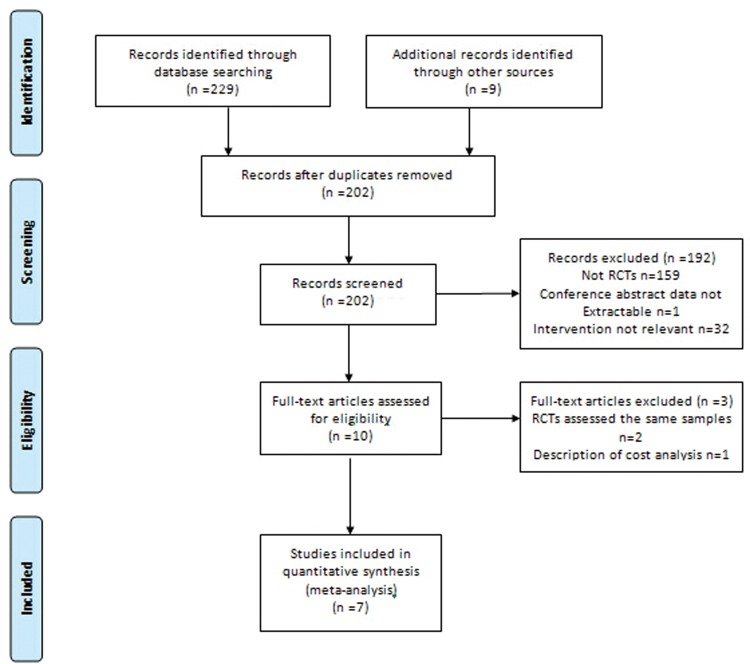
Flowchart. Flowchart of the selection of randomized controlled trials (RCTs) for the meta-analysis.

Seven different RCTs involving 735 study participants were fully analyzed. Fig. 1 shows the flow diagram used for study identification. We found three comparisons of OP with HoLEP [6, 7, 21]; four comparisons of OP with BEEP, including three trials using plasmakinetic enucleation of the prostate [22–24]; and one trial using bipolar plasma vaporization enucleation of the prostate [25]. OP was performed via a transvesical approach in all RCTs. The baseline characteristics of the included studies were individually extracted from each study and listed in Table 1. The inclusion and exclusion criteria were similar for most studies. Our meta-analysis reported good baseline characteristics with no significant differences, including age, prostate volume, PSA and preoperative micturition parameters such as Qmax, PVR, QoL, and IPSS. Of the studies included in this meta-analysis, one study reported the exact follow-up sample size in each group [22], 2 studies applied an intention-to-treat analysis [23, 24], and 4 studies applied the initial sample size to estimate the follow-up sample size [6, 7, 21, 25].

**Table 1:** Characteristics from the included RCTs comparing endoscopic enucleation of the prostate with open prostatectomy.

Reference	Publication year	Follow-up	Comparator	Trial size	Prostate	PSA	IPSS	Qmax	PVR	IIEF
		mo			volume, mL	ng/mL		mL/s	mL	
Kuntz et al. [7,34,35]	2002, 2004, 2008	1,3,6,12,18,24,36,48,60	HoLEP	60	114.6±21.6	NA	22.1±3.3	3.8±3.6	280.0±273.0	NA
			OP	60	113.0±19.2	NA	21.0±3.6	3.6±3.8	292.0±191.0	NA
Naspro et al. [6]	2006	1,3,12,24	HoLEP	41	113.3±35.3	6.3±3.5	20.1±5.8	7.8±3.4	NA	20.3±6.6
			OP	39	124.2±38.5	7.0±4.3	21.6±3.2	8.3±2.4	NA	21.1±5.3
Zhang et al. [21]	2007	3	HoLEP	32	139.6±26.4	NA	27.4±5.5	6.1±2.9	197.8±33.6	NA
			OP	28	157.2±35.1	NA	25.1±6.4	6.7±2.8	172.7±21.4	NA
Geavlete et al. [25]	2013	1,3,6,12,36	BPEP	70	132.6±50.0^a^	8.5±6.8	25.3±3.5	5.9±1.8	164.0±185.5	NA
			OP	70	129.7±48.8^a^	8.4±6.9	25.6±3.8	5.7±1.8	168.0±183.0	NA
Rao et al. [22]	2014	1,3,6,12	PKEP	43	116.2±32.4	4.8±2.2	24.8±3.1	5.8±2.0	83.4±11.8	20.6±3.1
			OP	40	110.2±32.1	4.5±2.1	24.5±3.6	5.9±2.3	81.4±15.7	20.3±3.4
Chen et al. [24]	2014	1,6,12,24,36,48,60,72	PKEP	80	110.0±20.7	2.9±0.9	25.6±3.3	4.0±2.2	240.0±170.4	22.0±3.0
			OP	80	114.5±17.8	3.1±0.7	25.7±3.3	4.0±2.0	249.0±163.0	22.0±3.7
Ou et al. [23]	2013	3,12	PKEP	47	132.2±36.9	5.9±0.7	23.2±5.7	5.9±2.1	89.6±52.7	NA
			OP	45	139.5±36.2	5.6±0.8	25.1±5.4	5.1±2.3	81.3±48.6	NA

^a^Unit: mL;

NA = not available; HoLEP = holmium laser enucleation of the prostate; BPEP = bipolar plasma vaporization enucleation of the prostate; PKEP = plasmakinetic enucleation of the prostate; IPSS = International Prostate Symptom Score; Qmax = maximum flow rate; QoL = quality of life; PVR = post-void residual urine volume; IIEF-5 = International Index of Erectile Function; mo = month.

### Risk of bias in the included studies

Table 2 summarizes the risk evaluation of bias. There were 6 high-quality RCTs and 1 low-quality RCT according to the Jadad scale [18, 26]. The nature of these studies made blinding impossible; thus, 6 studies received a score of 3, and 1 study received a score of 2 because it was unclear how random sequence generation had been carried out.

**Table 2:** Quality assessment of the included RCTs.

	Kuntz et al.	Naspro et al.	Zhang et al.	Geavlete et al.	Rao et al.	Chen et al.	Ou et al.
**Was the study described as randomized?**	1	1	1	1	1	1	1
**Was the method of randomization described and appropriate?**	1	1	0	1	1	1	1
**Was the study described as double blind?**	0	0	0	0	0	0	0
**Was the method of blinding described and appropriate?**	0	0	0	0	0	0	0
**Was there a description of withdrawals and dropouts?**	1	1	1	1	1	1	1
**Total**	3	3	2	3	3	3	3

### Perioperative outcomes

#### Operative time, catheterization time and hospital stay

In 7 trials reporting on the operative time, this factor was significantly longer in the EP group (16.21 [3.72, 28.70], P = 0.01). Nevertheless, 4 studies assessing BEEP vs OP showed no significant differences in the operative time (5.21 [-8.94, 19.35], P = 0.47), and 3 studies evaluating HoLEP demonstrated a significantly longer operative time compared with OP (32.15[8.87, 55.42], P = 0.007). However, the catheterization time (EP vs OP, -3.80 [-5.11, -2.48], P<0.00001) and hospital stay (EP vs OP, -4.93 [-5.96, -3.89], P<0.00001) were shorter compared with OP, and statistically significant differences were observed in the subgroup analyses. In addition, there was large heterogeneity among the studies.

#### Resected tissue weight

EP had numerically lower specimen weights compared with OP (-9.63 [-14.46, -4.81], P<0.00001). This result was also observed in the BEEP subgroup (-8.09[-12.90, -3.28], P = 0.001); however, no significant differences were noted in the HoLEP subgroup (-14.17[-28.33, -0.02], P = 0.05).

#### Decrease in hemoglobin

EP achieved a significantly smaller decrease in serum hemoglobin compared with OP (- 3.14 [-1.81, -0.47], P<0.00001). HoLEP (-0.95[-1.35, -0.56], P<0.00001) and BEEP (-1.22 [-2.12, -0.33], P<0.00001) also showed significantly smaller decreases in serum hemoglobin compared with OP. Table 3 shows the data on perioperative outcomes.

**Table 3:** Summary of perioperative outcomes

Outcome	No. of studies	Trial size EP/OP	WMD(95% CI)	P value	Heterogeneity		Favors
					I^2^	P value	
**Operative time, min**	/	/	/	/	/	/	/
**HoLEP vs OP**	6, 21, 35	133/127	32.15 [8.87, 55.42]*	0.01	93%	0.00	OP
**BEEP vs OP**	22–25	240/235	5.21 [-8.94, 19.35]*	0.47	93%	0.00	None
**EP vs OP total**	6, 21–25, 35	373/362	16.21 [3.72, 28.70]*	0.01	94%	0.00	OP
**Hemoglobin decrease, g/dL**	/	/	/	/	/	/	/
**HoLEP vs OP**	6, 35	101/99	-0.95 [-1.35, -0.56]*	0.00	0%	0.75	HoLEP
**BEEP vs OP**	22–25	240/235	-1.22 [-2.12, -0.33]*	0.01	97%	0.00	BEEP
**EP vs OP total**	6, 22–25, 35	341/334	-1.14 [-1.81, -0.47]*	0.00	96%	0.00	EP
**Resected prostate weight, g**	/	/	/	/	/	/	/
**HoLEP vs OP**	6, 21, 35	133/127	-14.17 [-28.33,-0.02]*	0.05	70%	0.03	None
**BEEP vs OP**	22–25	240/235	-8.09 [-12.90,-3.28]*	0.00	0%	0.91	OP
**EP vs OP total**	6, 21–25, 35	373/362	-9.63 [-14.46, -4.81]*	0.00	24%	0.24	OP
**Catheterization, days**	/	/	/	/	/	/	/
**HoLEP vs OP**	6, 21, 35	133/127	-3.83 [-7.17, -0.48]*	0.02	99%	0.00	HoLEP
**BEEP vs OP**	22–25	240/235	-3.78 [-4.51, -3.04]*	0.00	92%	0.00	BEEP
**EP vs OP total**	6, 21–25, 35	373/362	-3.80 [-5.11, -2.48]*	0.00	99%	0.00	EP
**Hospital stay, days**	/	/	/	/	/	/	/
**HoLEP vs OP**	6, 21, 35	133/127	-5.84 [-9.51, -2.17]*	0.00	99%	0.00	HoLEP
**BEEP vs OP**	22–25	240/235	-4.43 [-5.03, -3.84]*	0.00	85%	0.00	BEEP
**EP vs OP total**	6, 21–25, 35	373/362	-4.93 [-5.96, -3.89]*	0.00	97%	0.00	EP

*Using a random effect model;

EP = endoscopic enucleation of the prostate; OP = open prostatectomy; WMD = weighted mean difference; HoLEP = holmium laser enucleation of the prostate; BEEP = bipolar electrosurgical enucleation of the prostate.

### Postoperative outcomes

#### IPSS, Qmax, QoL, PVR, PSA and IIEF-5

There were no significant differences in the IPSS, Qmax, QoL and PVR between the groups at the 3-, 6- and 12-month postoperative follow-up, and no significant differences were observed in the subgroup analyses. EP was associated with higher IIEF-5 scores (1.00 [0.21, 1.78], p = 0.01) after 12 months. No differences were noted at the 3-, 6- and 24-month follow-up. Table 4 shows the data on postoperative outcomes.

**Table 4:** Summary of postoperative outcomes

Outcome	No. of studies	Trial size EP/OP	WMD(95% CI)	P value	Heterogeneity		Favors
					I^2^	P value	
**IPSS 3 mo HoLEP vs OP**	6, 21, 35	133/127	0.29 [-0.36, 0.93]	0.38	30%	0.24	None
**IPSS 3 mo BEEP vs OP**	22, 23, 25	160/155	0.15 [-0.45, 0.75]	0.63	0%	0.84	None
**IPSS 3 mo total**	6, 21–23, 25, 35	293/282	0.21 [-0.23, 0.65]	0.34	0%	0.65	None
**IPSS 6 mo HoLEP vs OP**	35	60/60	-0.40 [-1.50, 0.70]	0.48	/	/	None
**IPSS 6 mo BEEP vs OP**	22, 24, 25	191/189	0.04 [-0.52, 0.59]	0.90	0%	0.95	None
**IPSS 6 mo total**	22, 24, 25, 35	251/249	-0.05 [-0.55, 0.44]	0.83	0%	0.90	None
**IPSS 12 mo HoLEP vs OP**	6, 35	101/99	0.00 [-0.64, 0.65]	0.99	0%	0.97	None
**IPSS 12 mo BEEP vs OP**	22–25	237/233	-0.15 [-0.50, 0.21]	0.42	0%	0.98	None
**IPSS 12 mo total**	6, 22–25, 35	338/332	-0.11 [-0.42, 0.20]	0.48	0%	1.00	None
**Qmax 3 mo HoLEP vs OP**	6, 21, 35	133/127	-0.35 [-2.51, 1.81]*	0.79	21%	0.28	None
**Qmax 3 mo BEEP vs OP**	22, 23, 25	160/155	-0.70 [-3.08, 1.68]*	0.56	77%	0.01	None
**Qmax (mL/s) 3 mo total**	6, 21–23, 25, 35	293/282	-0.65 [-2.28, 0.98]*	0.44	64%	0.02	None
**Qmax 6 mo HoLEP vs OP**	35	60/60	2.90 [0.67, 5.13]	0.01	/	/	HoLEP
**Qmax 6 mo BEEP vs OP**	22, 24, 25	191/189	0.45 [-0.89, 1.78]	0.51	0%	0.92	None
**Qmax 6 mo total**	22, 24, 25, 35	251/249	1.09 [-0.05, 2.24]	0.06	17%	0.31	None
**Qmax 12 mo HoLEP vs OP**	6, 35	101/99	-1.53 [-3.40, 0.34]	0.11	0%	0.62	None
**Qmax 12 mo BEEP vs OP**	22–25	237/233	-0.31 [-1.40, 0.78]	0.58	0%	0.47	None
**Qmax 12 mo total**	6, 22–25, 35	338/332	-0.62 [-1.56, 0.32]	0.20	0%	0.55	None
**QoL 3 mo HoLEP vs OP**	6,21	73/67	0.24 [-0.06, 0.53]*	0.11	71%	0.06	None
**QoL 3 mo BEEP vs OP**	22, 23, 25	160/155	-0.15 [-0.37, 0.07]*	0.19	0%	0.75	None
**QoL3 mo total**	6, 21–23, 25	233/222	0.05 [-0.18, 0.27]*	0.69	63%	0.03	None
**QoL 6 mo HoLEP vs OP**	/	/	/	/	/	/	/
**QoL 6 mo BEEP vs OP**	22, 24, 25	191/189	-0.07 [-0.32, 0.19]	0.60	0%	0.94	None
**QoL 6 mo total**	22, 24, 25	191/189	-0.07 [-0.32, 0.19]	0.60	0%	0.94	None
**QoL 12 mo HoLEP vs OP**	6	41/39	-0.07[-0.46, 0.32]	0.72	/	/	None
**QoL 12 mo BEEP vs OP**	22–25	240/233	-0.08 [-0.25, 0.09]	0.38	0%	0.74	None
**QoL 12 mo total**	6, 22–25	281/272	-0.08 [-0.23, 0.08]	0.35	0%	0.87	None
**PVR 3 mo HoLEP vs OP**	21, 35	92/88	-0.75 [-10.93, 9.43]*	0.88	83%	0.02	None
**PVR 3 mo BEEP vs OP**	22, 23, 25	160/155	-0.46 [-2.27, 1.35]*	0.62	0%	0.89	None
**PVR (mL) 3 mo total**	21–23, 25, 35	252/243	-0.47 [-3.32, 2.38]*	0.75	35%	0.19	None
**PVR 6 mo HoLEP vs OP**	35	60/60	2.30 [-0.87, 5.47]	0.16	/	/	None
**PVR 6 mo BEEP vs OP**	22, 24, 25	191/189	-0.29 [-1.64, 1.07]	0.68	0%	0.99	None
**PVR 6 mo total**	22, 24, 25, 35	251/249	0.11 [-1.13, 1.36]	0.86	0%	0.54	None
**PVR 12 mo HoLEP vs OP**	35	60/60	-0.60 [-5.85, 4.65]	0.82	/	/	None
**PVR 12 mo BEEP vs OP**	22–25	237/233	-0.20 [-1.39, 0.99]	0.74	0%	0.61	None
**PVR 12 mo total**	22–25, 35	297/293	-0.22 [-1.38, 0.94]	0.71	0%	0.76	None
**IIEF-5 3 mo EP vs OP**	6, 22	84/79	0.47 [-0.64, 1.59]	0.41	0%	0.68	None
**IIEF-5 6 mo EP vs OP**	6, 22, 24	162/158	-0.44[-2.03, 1.14]*	0.58	61%	0.08	None
**IIEF-5 12 mo EP vs OP**	6, 22, 24	161/157	1.00[0.21, 1.78]	0.01	9%	0.33	EP
**IIEF-5 24 mo EP vs OP**	6, 24	121/119	0.89 [-0.01, 1.80]	0.05	0%	0.62	None

*Using a random effect model;

EP = endoscopic enucleation of the prostate; OP = open prostatectomy; WMD = weighted mean difference; HoLEP = holmium laser enucleation of the prostate; BEEP = bipolar electrosurgical enucleation of the prostate; IPSS = International Prostate Symptom Score; Qmax = maximum flow rate; QoL = quality of life; PVR = post-void residual urine volume; IIEF-5 = International Index of Erectile Function; mo = month.

#### Complications

Table 5 displays our meta-analysis results of complications after surgery. The need for blood transfusion in the EP group was significantly lower than that in the OP group (0.22 [0.10, 0.47], P = 0.0001). No statistically significant difference was observed between the EP and OP groups with respect to recatheterization, urinary tract infection, urinary incontinence, bladder-neck/urethral strictures or reintervention.

**Table 5:** Summary of complications.

Outcome	No. of studies	Trial size EP/OP	RR(95% CI)	P value	Heterogeneity		Favors
					I^2^	P value	
**Blood transfusion**	/	/	/	/	/	/	/
**HoLEP vs OP**	6, 34	101/99	0.16 [0.04, 0.58]	0.01	0%	0.32	HoLEP
**BEEP vs OP**	22–25	240/235	0.27 [0.10, 0.72]	0.01	16%	0.31	BEEP
**EP vs OP total**	6, 22–25, 34	341/334	0.22 [0.10, 0.47]	0.00	0%	0.42	EP
**Recatheterization**	/	/	/	/	/	/	/
**HoLEP vs OP**	6, 34	101/99	1.56 [0.53, 4.62]	0.42	0%	0.44	None
**BEEP vs OP**	22–25	240/235	0.39 [0.12, 1.22]	0.10	17%	0.30	None
**EP vs OP total**	6, 22–25, 34	341/334	0.78 [0.37, 1.63]	0.51	25%	0.26	None
**Urinary tract infection**	/	/	/	/	/	/	/
**HoLEP vs OP**	/	/	/	/	/	/	/
**BEEP vs OP**	22–25	240/235	0.60 [0.31, 1.18]	0.14	0%	0.93	None
**EP vs OP total**	22–25	240/235	0.60 [0.31, 1.18]	0.14	0%	0.93	None
**Urinary incontinence**	/	/	/	/	/	/	/
**HoLEP vs OP**	6, 21	73/67	0.86 [0.53, 1.40]*	0.55	0%	0.40	None
**BEEP vs OP**	22–25	162/228	1.45 [0.19, 11.25]*	0.72	83%	0.00	None
**EP vs OP total**	6, 21–25	235/295	1.35 [0.42, 4.37]*	0.62	85%	0.00	None
**BNC/urethral strictures**	/	/	/	/	/	/	/
**HoLEP vs OP**	6, 21, 34	133/127	0.78 [0.24, 2.49]	0.67	0%	0.91	None
**BEEP vs OP**	22–25	234/228	0.69 [0.31, 1.54]	0.36	0%	0.47	None
**EP vs OP total**	6, 21–25, 34	367/355	0.71 [0.37, 1.39]	0.32	0%	0.84	None
**Reintervention**	/	/	/	/	/	/	/
**HoLEP vs OP**	6, 7, 21, 34	133/127	1.06 [0.49, 2.29]	0.89	0%	0.96	None
**BEEP vs OP**	22–25	234/228	0.71 [0.33, 1.53]	0.38	0%	0.46	None
**EP vs OP total**	6, 7, 21–25, 34	367/355	0.86 [0.50, 1.48]	0.58	0%	0.81	None

*Using a random effect model;

EP = endoscopic enucleation of the prostate; OP = open prostatectomy; RR = risk ratio; HoLEP = holmium laser enucleation of prostate; BEEP = bipolar electrosurgical enucleation of the prostate; BNC = bladder neck contracture.

#### Publication bias analyses

We also tested for possible publication bias in all of the evaluated comparisons. No clear publication bias was apparent.

## Discussion

The first surgical enucleation for BPH was reported by Freyer in 1919 [27]. This method continues to be associated with a low re-treatment rate and more complete procedure for the removal of prostatic tissue of any size. However, the disadvantages of OP include mortality (<0.25%), blood transfusion (7–14%) [7, 28], urinary incontinence (≤10%) and bladder neck stenosis or urethral stricture (6%) [6, 7]. Despite the occurrence of more intraoperative bleeding, longer catheterization times, and longer hospital stays, OP is still used for 3% of the prostatectomies in the United States [29], 14% in France [30], 12% in Sweden [31] and 40% in Israel [32]. Consequently, research into alternative surgical treatments (for large prostates) with similar efficacies but minimal complications has continued.

In this review, 7 contemporary RCTs published between 2002 and 2014 that included 735 patients with prostate volumes >70 mL and compared EP with OP over a maximum follow-up of 6 years [24] were analyzed. We focused on perioperative variables, postoperative outcomes and complications. Two energy sources, the holmium laser and bipolar energy systems, were applied in our review. Thus, perioperative and postoperative outcomes and complications must be cautiously and separately estimated for each system. We performed subgroup analyses to test for possible differences between HoLEP and bipolar electrosurgical enucleation of the prostate.

In our analysis, the data revealed that EP might have a more desirable perioperative profile. A smaller decrease in hemoglobin was observed following EP because the superiority of this reduction in blood loss might be supported by the excellent coagulation technique used in EP [33]. Less bleeding in EP led to a reduced catheterization time, and the reduced catheterization time resulted in a shorter hospital stay compared to OP. Statistically significant differences were also observed in the subgroup analyses. Although 4 trials reported that the resected tissue weights between the two groups were not significantly different [23–25, 34], the pooled data revealed that EP yielded lower specimen weights compared with OP. In the subgroup analysis, BEEP yielded lower specimen weights than OP, but HoLEP showed no significant difference compared with OP. After the whole adenoma was nearly dissected from the capsule, the enucleated lobes were fragmented by a mechanical tissue morcellator in three trials [6, 21, 25], and fragmentation of the subtotally enucleated lobes was performed by traditional electrocautery loop resection in the other three trials [22–24]. One trial used traditional electrocautery loop resection in the first 50 patients and mechanical tissue morcellator in the last 10 patients [7]. The reason that EP has lower specimen weights than OP might be due to specimen weight loss during vaporized resection or the procedure that uses a mechanical tissue morcellator. The operation time was almost 16 min longer in EP. In the subgroup analyses, the operation duration of BEEP was similar to that of OP; however, the operation duration of HoLEP was longer compared with OP. All three trials assessing HoLEP reported longer operation time [6, 21, 35], and a longer operation time was also reported with plasmakinetic enucleation of the prostate [24]. In the other three studies using the bipolar energy system, no difference in the operative time was detected [22, 23, 25]. This can be explained by the steep learning curve required for HoLEP [36]. In addition, there was a significant amount of heterogeneity among the studies. Only one study clearly stated that both procedures were performed by a highly experienced surgeon [22], and another study showed that all procedures in both groups were conducted by two senior staff urologists [6]. However, it was not clearly stated whether both arms were performed by the same surgeon in the other 5 trials. Tissue morcellation was used for the enucleated tissues in 4 trials [6, 7, 21, 25]. In our study, the prostate sizes between groups were similar; thus, the extra time necessary for morcellation of the enucleated tissues, difficulties in the operation, different clinical practices among different countries, and several operator-dependent and technical characteristics might contribute to this significant heterogeneity. In general, EP offered several advantages over OP in terms of the catheterization time, hospital stay length, and hemoglobin decrease, whereas OP was superior in terms of the operation time and resected tissue weight.

Only three trials could be included in our analysis of the IIEF-5 score [6, 22, 24]. The pooled data showed an improvement at the 12-month follow-up; this can be attributed to the precise resection, which made it possible to preserve the tissue around the verumontanum. The weighted mean difference for EP vs OP was 0.89 [-0.01, 1.80], P = 0.05 at the 24-month follow-up, which was likely due to the limited sample size. There was a non-significant trend at the 3- and 6-month follow-up. Due to the lack of data, we did not perform subgroup analyses on sexual function.

EP was associated with improvements similar to those of OP in terms of the IPSS, Qmax, QoL, PVR and PSA at the 3-, 6- and 12-month follow-up. EP was found to be equivalent to OP at the 12-month follow-up for men with large prostates. Additionally, no significant differences were found in the HoLEP and BEEP subgroup analyses. Three trials included in this review that provided longer-term follow-ups, ranging from 12 to 72 months, also supported this result [6, 7, 24], and similar data were detected concerning the prostate volume after surgery at the 6- and 12-month follow-up in one trial [25].

This pooled analysis of the RCTs revealed that EP has distinct advantages in terms of the need for blood transfusion; this is likely due to blockage of the blood supply to the prostatic adenoma and the use of excellent coagulation methods to control intraoperative bleeding in this minimally invasive technique [33]. BEEP and HoLEP also supported this result in the subgroup analyses. There were no significant differences in the complications of recatheterization, urinary tract infection, urinary incontinence, bladder-neck/urethral strictures and reintervention, and no differences were observed in the subgroup analyses.

## Conclusions

This meta-analysis revealed statistically comparable efficacy and safety for EP vs OP, although only a limited number of RCTs with relatively limited follow-up are available. EP had an efficacy similar to that of OP in terms of the IPSS, Qmax, QoL, PVR and PSA and offered several advantages over OP in terms of the catheterization time, hospital stay, hemoglobin decrease, blood transfusion and IIEF-5 score. By contrast, OP was superior in terms of the operation time and the resected tissue weight. Furthermore, no differences were evident regarding the rates of complications such as recatheterization, urinary tract infection, urinary incontinence, bladder-neck/urethral strictures and reintervention. In general, EP is an effective and safe minimally invasive option for the treatment of large prostates.

## Supporting Information

S1 Fig Forest plot for operative time. EP = endoscopic enucleation of the prostate; OP = open prostatectomy; CI = confidence interval.
(TIF)

S2 Fig Forest plot for catheterization. EP = endoscopic enucleation of the prostate; OP = open prostatectomy; CI = confidence interval.
(TIF)

S3 Fig Forest plot for hospital stay. EP = endoscopic enucleation of the prostate; OP = open prostatectomy; CI = confidence interval.
(TIF)

S4 Fig Forest plot for resected prostate weight. EP = endoscopic enucleation of the prostate; OP = open prostatectomy; CI = confidence interval.
(TIF)

S5 Fig Forest plot for hemoglobin decrease. EP = endoscopic enucleation of the prostate; OP = open prostatectomy; CI = confidence interval.
(TIF)

S6 Fig Forest plot for IPSS at 3-month. EP = endoscopic enucleation of the prostate; OP = open prostatectomy; IPSS = International Prostate Symptom Score; CI = confidence interval.
(TIF)

S7 Fig Forest plot for IPSS at 6-month. EP = endoscopic enucleation of the prostate; OP = open prostatectomy; IPSS = International Prostate Symptom Score; CI = confidence interval.
(TIF)

S8 Fig Forest plot for IPSS at 12-month. EP = endoscopic enucleation of the prostate; OP = open prostatectomy; IPSS = International Prostate Symptom Score; CI = confidence interval.
(TIF)

S9 Fig Forest plot for Qmax at 3-month. EP = endoscopic enucleation of the prostate; OP = open prostatectomy; Qmax = maximum flow rate; CI = confidence interval.
(TIF)

S10 Fig Forest plot for Qmax at 6-month. EP = endoscopic enucleation of the prostate; OP = open prostatectomy; Qmax = maximum flow rate; CI = confidence interval.
(TIF)

S11 Fig Forest plot for Qmax at 12-month. EP = endoscopic enucleation of the prostate; OP = open prostatectomy; Qmax = maximum flow rate; CI = confidence interval.
(TIF)

S12 Fig Forest plot for QoL at 3-month. EP = endoscopic enucleation of the prostate; OP = open prostatectomy; QoL = quality of life; CI = confidence interval.
(TIF)

S13 Fig Forest plot for QoL at 6-month. EP = endoscopic enucleation of the prostate; OP = open prostatectomy; QoL = quality of life; CI = confidence interval.
(TIF)

S14 Fig Forest plot for QoL at 12-month. EP = endoscopic enucleation of the prostate; OP = open prostatectomy; QoL = quality of life; CI = confidence interval.
(TIF)

S15 Fig Forest plot for PVR at 3-month. EP = endoscopic enucleation of the prostate; OP = open prostatectomy; PVR = post-void residual urine volume; CI = confidence interval.
(TIF)

S16 Fig Forest plot for PVR at 6-month. EP = endoscopic enucleation of the prostate; OP = open prostatectomy; PVR = post-void residual urine volume; CI = confidence interval.
(TIF)

S17 Fig Forest plot for PVR at 12-month. EP = endoscopic enucleation of the prostate; OP = open prostatectomy; PVR = post-void residual urine volume; CI = confidence interval.
(TIF)

S18 Fig Forest plot for IIEF-5 at 3-month. EP = endoscopic enucleation of the prostate; OP = open prostatectomy; IIEF-5 = International Index of Erectile Function; CI = confidence interval.
(TIF)

S19 Fig Forest plot for IIEF-5 at 6-month. EP = endoscopic enucleation of the prostate; OP = open prostatectomy; IIEF-5 = International Index of Erectile Function; CI = confidence interval.
(TIF)

S20 Fig Forest plot for IIEF-5 at 12-month. EP = endoscopic enucleation of the prostate; OP = open prostatectomy; IIEF-5 = International Index of Erectile Function; CI = confidence interval.
(TIF)

S21 Fig Forest plot for IIEF-5 at 24-month. EP = endoscopic enucleation of the prostate; OP = open prostatectomy; IIEF-5 = International Index of Erectile Function; CI = confidence interval.
(TIF)

S22 Fig Forest plot for blood transfusion. EP = endoscopic enucleation of the prostate; OP = open prostatectomy; CI = confidence interval.
(TIF)

S23 Fig Forest plot for recatheterization. EP = endoscopic enucleation of the prostate; OP = open prostatectomy; CI = confidence interval.
(TIF)

S24 Fig Forest plot for urinary tract infection. EP = endoscopic enucleation of the prostate; OP = open prostatectomy; CI = confidence interval.
(TIF)

S25 Fig Forest plot for urinary incontinence. EP = endoscopic enucleation of the prostate; OP = open prostatectomy; CI = confidence interval.
(TIF)

S26 Fig Forest plot for BNC/urethral strictures. EP = endoscopic enucleation of the prostate; OP = open prostatectomy; BNC = bladder-neck contracture; CI = confidence interval.
(TIF)

S27 Fig Forest plot for reintervention. EP = endoscopic enucleation of the prostate; OP = open prostatectomy; CI = confidence interval.
(TIF)

S1 File Search strategy. Search strategy used for electronic databases, including PubMed/Medline, EMBASE, The Cochrane Library and Web of Science.
(DOCX)

S2 File FigShare DOIs. Here's the DOIs necessary to access my data in the Table 3, Table 4 and Table 5.
(DOCX)

S1 PRISMA checklist From: Moher D, Liberati A, Tetzlaff J, Altman DG, The PRISMA Group (2009). (DOC)
