# Supplementary figures and images for: Endoscopic Enucleation versus Open Prostatectomy for Treating Large Benign Prostatic Hyperplasia: A Meta-Analysis of Randomized Controlled Trials
Source: PLoS One. 2015 Mar 31;10(3):e0121265. doi: 10.1371/journal.pone.0121265 (PMC4380430; doi:10.1371/journal.pone.0121265)

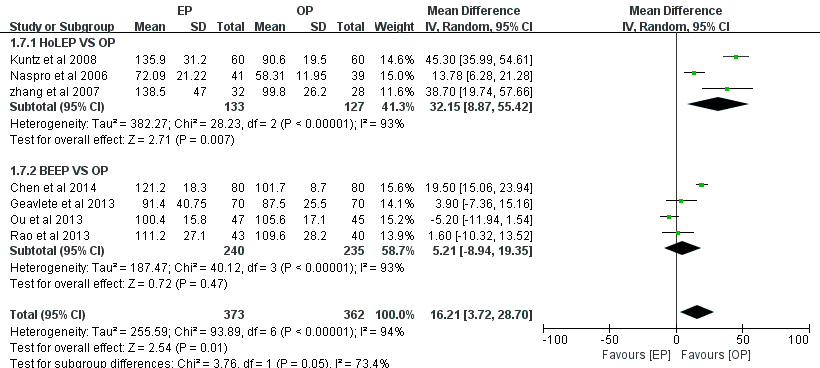

Supplement: S1 Fig — EP = endoscopic enucleation of the prostate; OP = open prostatectomy; CI = confidence interval. (TIF) [file pone.0121265.s001.tif]

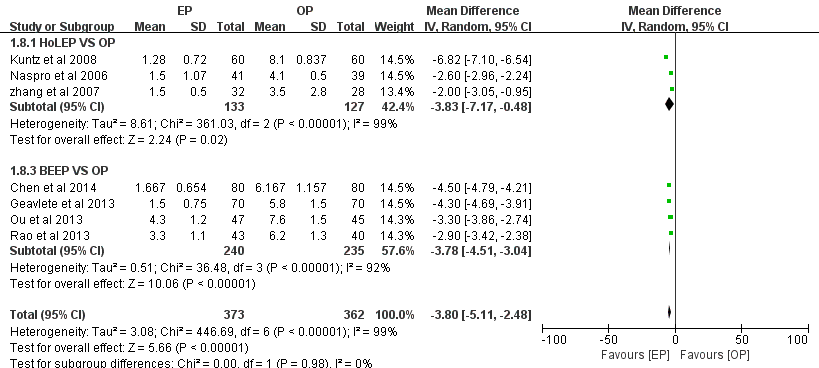

Supplement: S2 Fig — EP = endoscopic enucleation of the prostate; OP = open prostatectomy; CI = confidence interval. (TIF) [file pone.0121265.s002.tif]

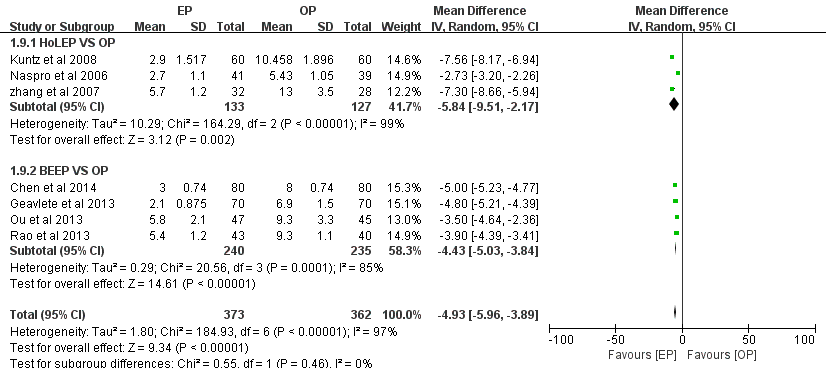

Supplement: S3 Fig — EP = endoscopic enucleation of the prostate; OP = open prostatectomy; CI = confidence interval. (TIF) [file pone.0121265.s003.tif]

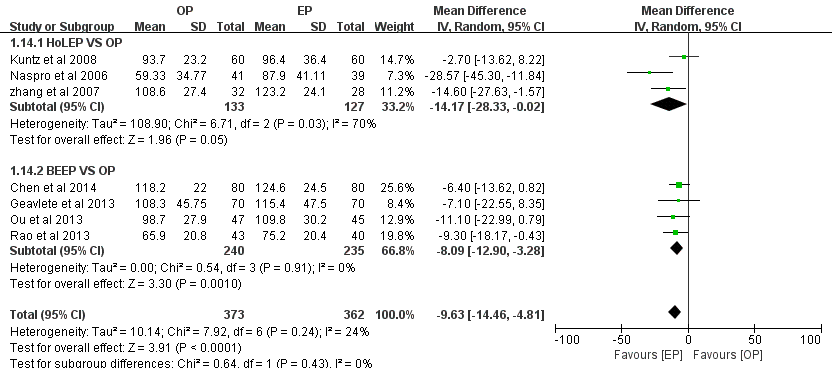

Supplement: S4 Fig — EP = endoscopic enucleation of the prostate; OP = open prostatectomy; CI = confidence interval. (TIF) [file pone.0121265.s004.tif]

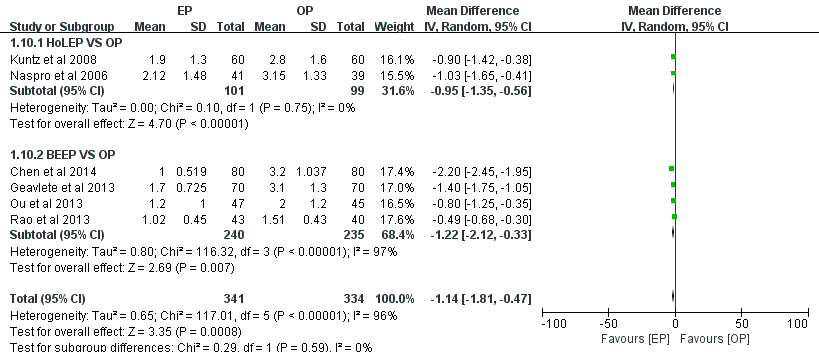

Supplement: S5 Fig — EP = endoscopic enucleation of the prostate; OP = open prostatectomy; CI = confidence interval. (TIF) [file pone.0121265.s005.tif]

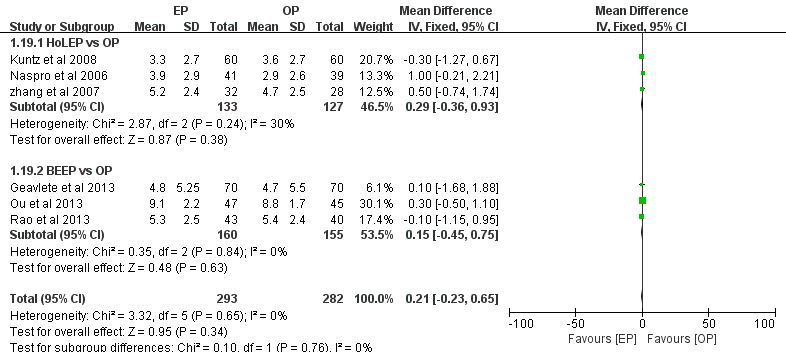

Supplement: S6 Fig — EP = endoscopic enucleation of the prostate; OP = open prostatectomy; IPSS = International Prostate Symptom Score; CI = confidence interval. (TIF) [file pone.0121265.s006.tif]

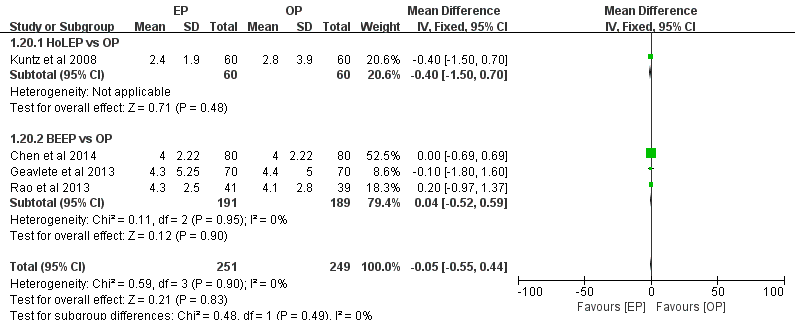

Supplement: S7 Fig — EP = endoscopic enucleation of the prostate; OP = open prostatectomy; IPSS = International Prostate Symptom Score; CI = confidence interval. (TIF) [file pone.0121265.s007.tif]

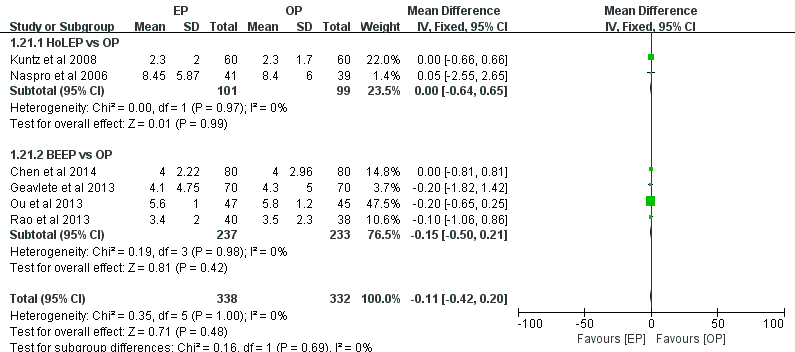

Supplement: S8 Fig — EP = endoscopic enucleation of the prostate; OP = open prostatectomy; IPSS = International Prostate Symptom Score; CI = confidence interval. (TIF) [file pone.0121265.s008.tif]

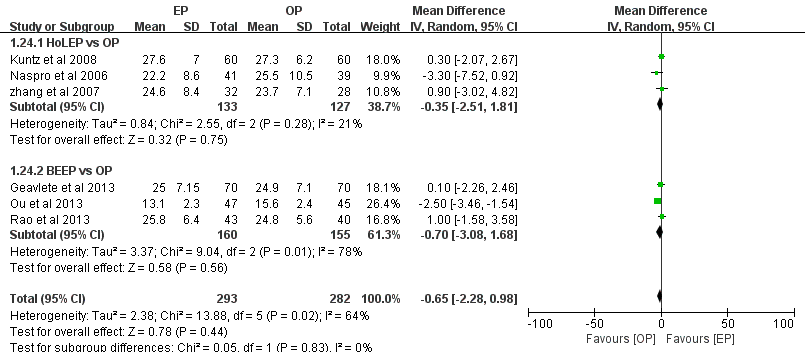

Supplement: S9 Fig — EP = endoscopic enucleation of the prostate; OP = open prostatectomy; Qmax = maximum flow rate; CI = confidence interval. (TIF) [file pone.0121265.s009.tif]

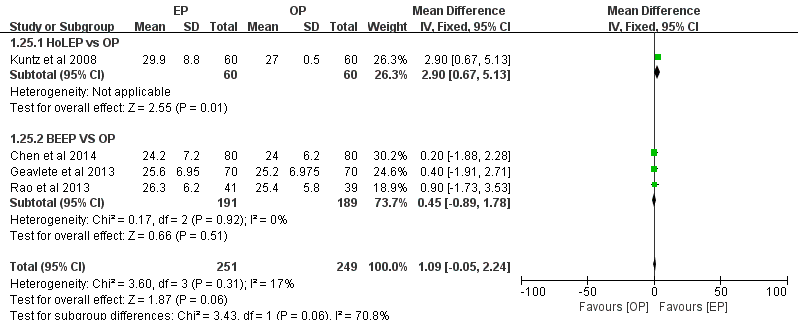

Supplement: S10 Fig — EP = endoscopic enucleation of the prostate; OP = open prostatectomy; Qmax = maximum flow rate; CI = confidence interval. (TIF) [file pone.0121265.s010.tif]

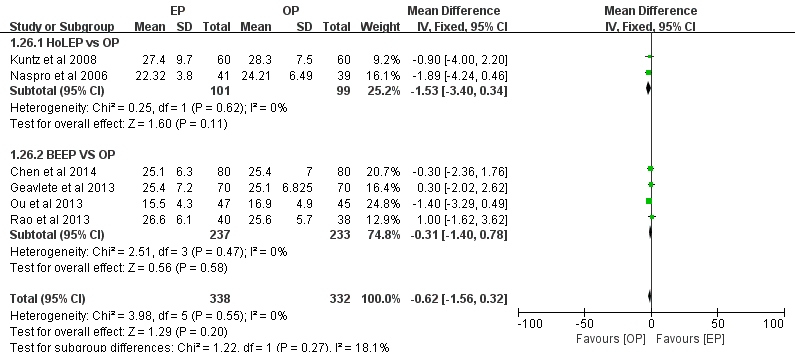

Supplement: S11 Fig — EP = endoscopic enucleation of the prostate; OP = open prostatectomy; Qmax = maximum flow rate; CI = confidence interval. (TIF) [file pone.0121265.s011.tif]

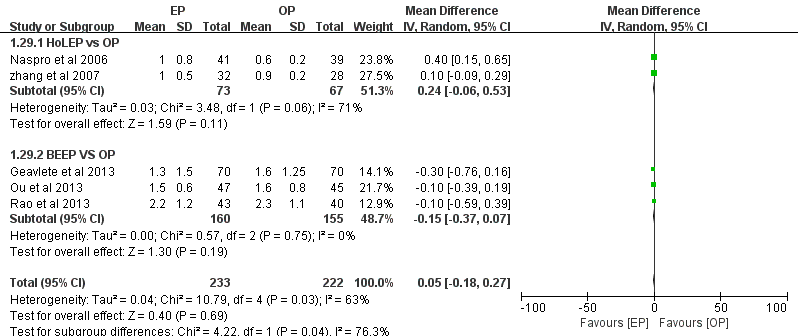

Supplement: S12 Fig — EP = endoscopic enucleation of the prostate; OP = open prostatectomy; QoL = quality of life; CI = confidence interval. (TIF) [file pone.0121265.s012.tif]

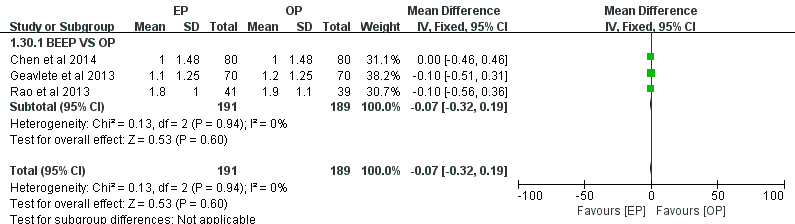

Supplement: S13 Fig — EP = endoscopic enucleation of the prostate; OP = open prostatectomy; QoL = quality of life; CI = confidence interval. (TIF) [file pone.0121265.s013.tif]

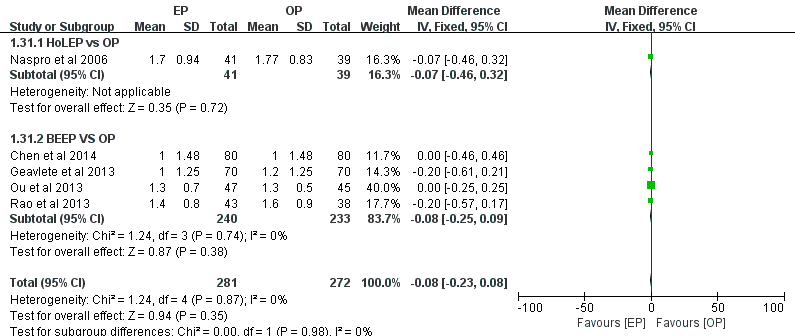

Supplement: S14 Fig — EP = endoscopic enucleation of the prostate; OP = open prostatectomy; QoL = quality of life; CI = confidence interval. (TIF) [file pone.0121265.s014.tif]

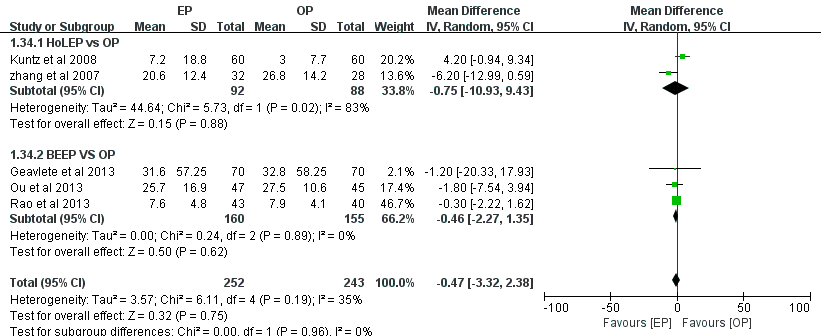

Supplement: S15 Fig — EP = endoscopic enucleation of the prostate; OP = open prostatectomy; PVR = post-void residual urine volume; CI = confidence interval. (TIF) [file pone.0121265.s015.tif]

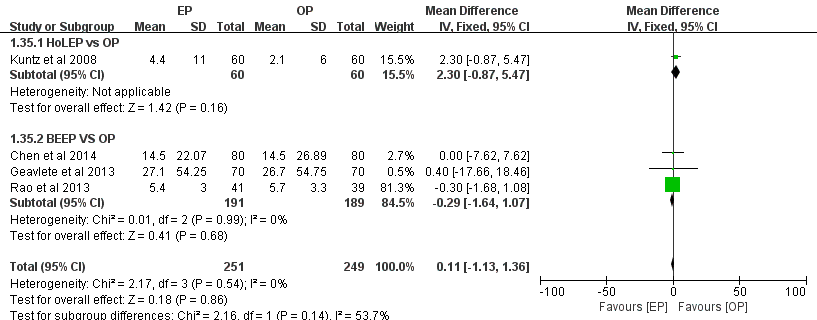

Supplement: S16 Fig — EP = endoscopic enucleation of the prostate; OP = open prostatectomy; PVR = post-void residual urine volume; CI = confidence interval. (TIF) [file pone.0121265.s016.tif]

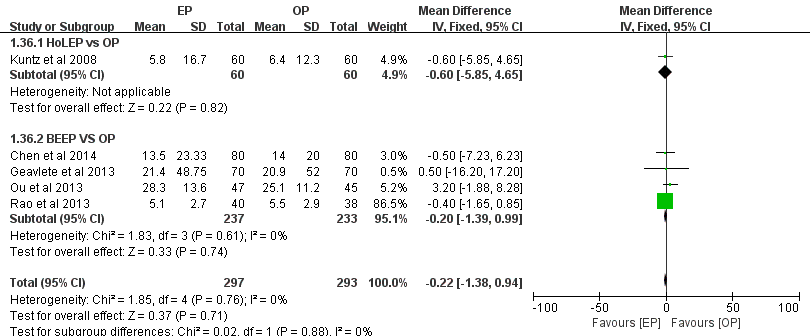

Supplement: S17 Fig — EP = endoscopic enucleation of the prostate; OP = open prostatectomy; PVR = post-void residual urine volume; CI = confidence interval. (TIF) [file pone.0121265.s017.tif]

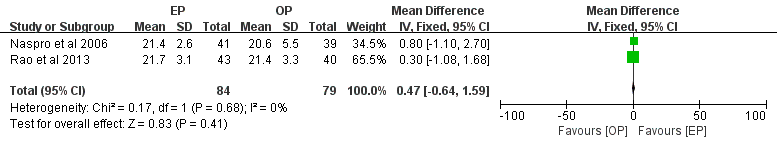

Supplement: S18 Fig — EP = endoscopic enucleation of the prostate; OP = open prostatectomy; IIEF-5 = International Index of Erectile Function; CI = confidence interval. (TIF) [file pone.0121265.s018.tif]

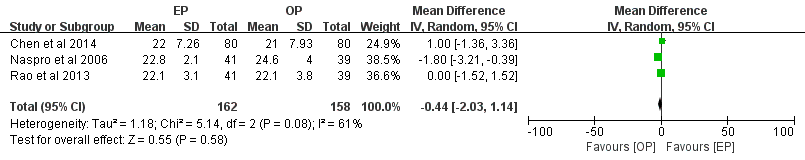

Supplement: S19 Fig — EP = endoscopic enucleation of the prostate; OP = open prostatectomy; IIEF-5 = International Index of Erectile Function; CI = confidence interval. (TIF) [file pone.0121265.s019.tif]

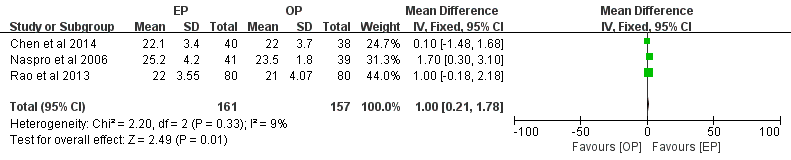

Supplement: S20 Fig — EP = endoscopic enucleation of the prostate; OP = open prostatectomy; IIEF-5 = International Index of Erectile Function; CI = confidence interval. (TIF) [file pone.0121265.s020.tif]

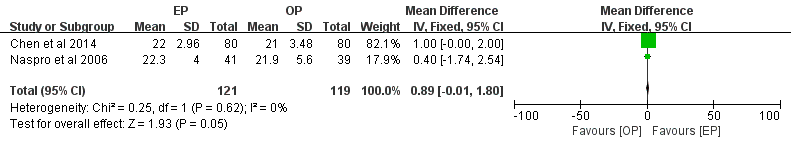

Supplement: S21 Fig — EP = endoscopic enucleation of the prostate; OP = open prostatectomy; IIEF-5 = International Index of Erectile Function; CI = confidence interval. (TIF) [file pone.0121265.s021.tif]

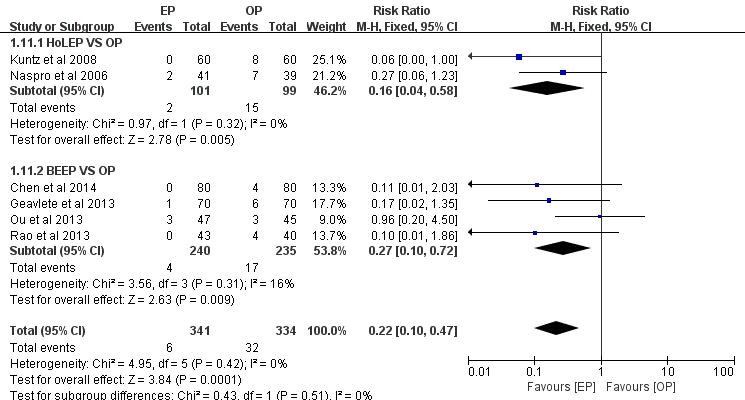

Supplement: S22 Fig — EP = endoscopic enucleation of the prostate; OP = open prostatectomy; CI = confidence interval. (TIF) [file pone.0121265.s022.tif]

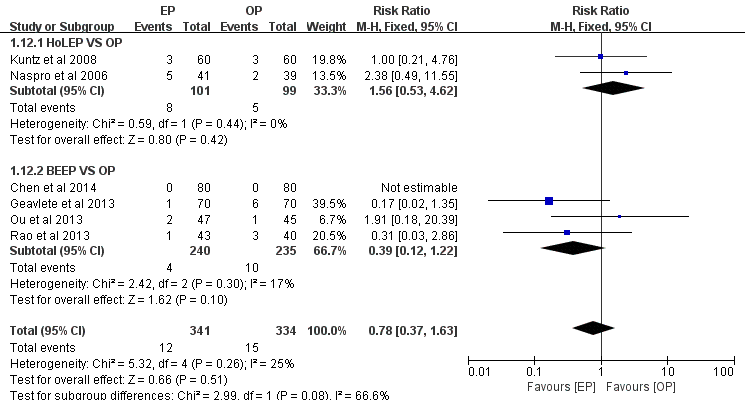

Supplement: S23 Fig — EP = endoscopic enucleation of the prostate; OP = open prostatectomy; CI = confidence interval. (TIF) [file pone.0121265.s023.tif]

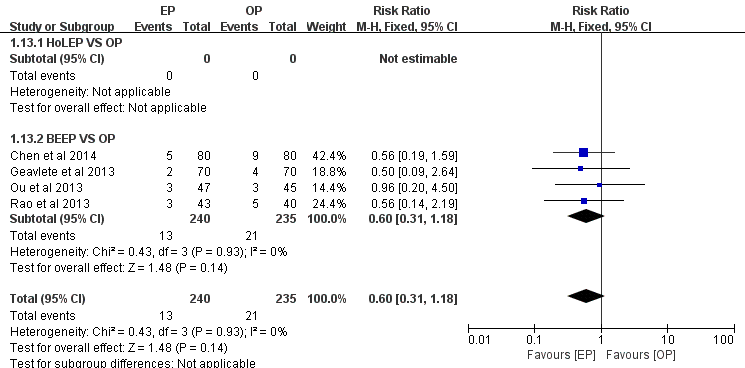

Supplement: S24 Fig — EP = endoscopic enucleation of the prostate; OP = open prostatectomy; CI = confidence interval. (TIF) [file pone.0121265.s024.tif]

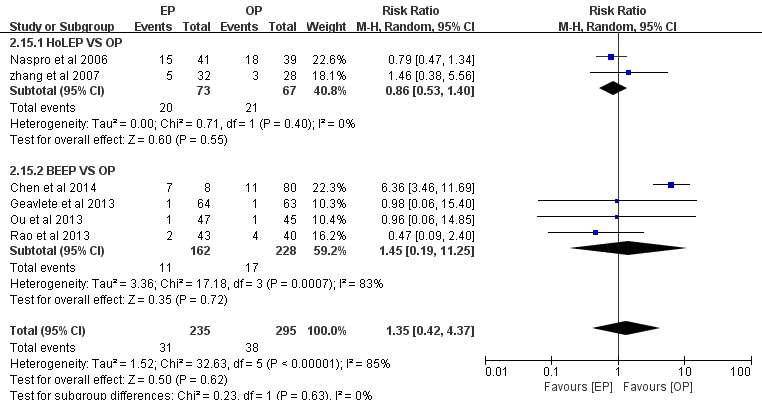

Supplement: S25 Fig — EP = endoscopic enucleation of the prostate; OP = open prostatectomy; CI = confidence interval. (TIF) [file pone.0121265.s025.tif]

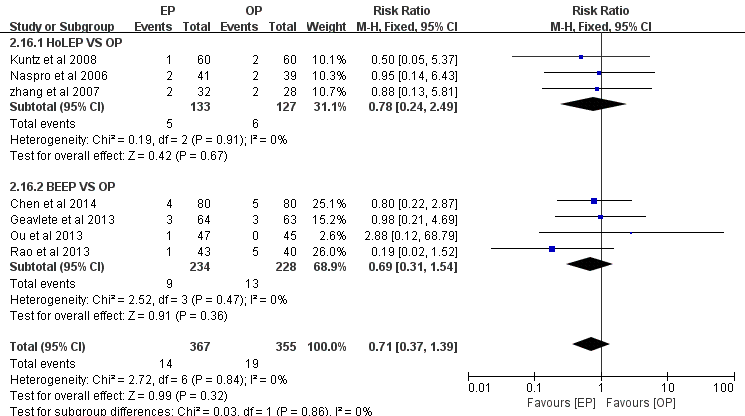

Supplement: S26 Fig — EP = endoscopic enucleation of the prostate; OP = open prostatectomy; BNC = bladder-neck contracture; CI = confidence interval. (TIF) [file pone.0121265.s026.tif]

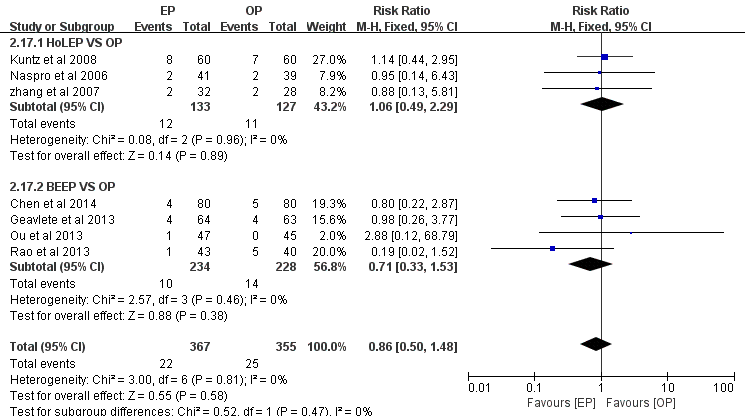

Supplement: S27 Fig — EP = endoscopic enucleation of the prostate; OP = open prostatectomy; CI = confidence interval. (TIF) [file pone.0121265.s027.tif]
