# Supplementary material for: Endoscopic Enucleation versus Open Prostatectomy for Treating Large Benign Prostatic Hyperplasia: A Meta-Analysis of Randomized Controlled Trials
Source: PLoS One. 2015 Mar 31;10(3):e0121265. doi: 10.1371/journal.pone.0121265 (PMC4380430; doi:10.1371/journal.pone.0121265)
Supplement: S1 File — Search strategy used for electronic databases, including PubMed/Medline, EMBASE, The Cochrane Library and Web of Science. (DOCX) [file pone.0121265.s028.docx]

**Appendix A. Search strategy protocols used for each electronic database**

(1) PubMed:

(enucleation[All Fields] AND ((open[All Fields] AND ("prostatectomy"[MeSH Terms] OR "prostatectomy"[All Fields])) OR ("prostatectomy"[MeSH Terms] OR "prostatectomy"[All Fields] OR ("transvesical"[All Fields] AND "prostatectomy"[All Fields]) OR "transvesical prostatectomy"[All Fields]) OR (transvesical[All Fields] AND open[All Fields] AND ("prostatectomy"[MeSH Terms] OR "prostatectomy"[All Fields])) OR (transvesical[All Fields] AND open[All Fields] AND enucleation[All Fields]) OR ("prostatectomy"[MeSH Terms] OR "prostatectomy"[All Fields] OR ("retropubic"[All Fields] AND "prostatectomy"[All Fields]) OR "retropubic prostatectomy"[All Fields]) OR (retropubic[All Fields] AND adenomectomy[All Fields]) OR adenomectomy[All Fields] OR ("prostatectomy"[MeSH Terms] OR "prostatectomy"[All Fields]) OR (prostatectom[All Fields] OR prostatectom'ia[All Fields] OR prostatectom'ias[All Fields] OR prostatectomaa[All Fields] OR prostatectomaas[All Fields] OR prostatectomi[All Fields] OR prostatectomi'ak[All Fields] OR prostatectomia[All Fields] OR prostatectomiaban[All Fields] OR prostatectomiak[All Fields] OR prostatectomiaradical[All Fields] OR prostatectomias[All Fields] OR prostatectomiaval[All Fields] OR prostatectomic[All Fields] OR prostatectomie[All Fields] OR prostatectomies[All Fields] OR prostatectomis'e[All Fields] OR prostatectomis'es[All Fields] OR prostatectomised[All Fields] OR prostatectomises[All Fields] OR prostatectomists[All Fields] OR prostatectomizados[All Fields] OR prostatectomize[All Fields] OR prostatectomized[All Fields] OR prostatectomizzati[All Fields] OR prostatectomizzato[All Fields] OR prostatectomy[All Fields] OR prostatectomy'[All Fields] OR prostatectomy's[All Fields] OR prostatectomyjsm[All Fields] OR prostatectomyperioperative[All Fields] OR prostatectomyu[All Fields]))) AND (("prostatic hyperplasia"[MeSH Terms] OR ("prostatic"[All Fields] AND "hyperplasia"[All Fields]) OR "prostatic hyperplasia"[All Fields]) OR ("prostatic hyperplasia"[MeSH Terms] OR ("prostatic"[All Fields] AND "hyperplasia"[All Fields]) OR "prostatic hyperplasia"[All Fields] OR ("prostate"[All Fields] AND "hypertrophy"[All Fields]) OR "prostate hypertrophy"[All Fields]) OR (("prostate"[MeSH Terms] OR "prostate"[All Fields] OR "prostatic"[All Fields]) AND ("hypertrophy"[MeSH Terms] OR "hypertrophy"[All Fields] OR "enlargement"[All Fields])) OR ("urinary bladder neck obstruction"[MeSH Terms] OR ("urinary"[All Fields] AND "bladder"[All Fields] AND "neck"[All Fields] AND "obstruction"[All Fields]) OR "urinary bladder neck obstruction"[All Fields]) OR ("prostatic hyperplasia"[MeSH Terms] OR ("prostatic"[All Fields] AND "hyperplasia"[All Fields]) OR "prostatic hyperplasia"[All Fields] OR ("prostate"[All Fields] AND "adenoma"[All Fields]) OR "prostate adenoma"[All Fields]) OR ("prostatic hyperplasia"[MeSH Terms] OR ("prostatic"[All Fields] AND "hyperplasia"[All Fields]) OR "prostatic hyperplasia"[All Fields] OR ("benign"[All Fields] AND "prostatic"[All Fields] AND "hyperplasia"[All Fields]) OR "benign prostatic hyperplasia"[All Fields]) OR ("urinary bladder neck obstruction"[MeSH Terms] OR ("urinary"[All Fields] AND "bladder"[All Fields] AND "neck"[All Fields] AND "obstruction"[All Fields]) OR "urinary bladder neck obstruction"[All Fields] OR ("bladder"[All Fields] AND "outlet"[All Fields] AND "obstruction"[All Fields]) OR "bladder outlet obstruction"[All Fields]))

(2)The Cochrane Library:

(prostatic hyperplasia OR prostate hypertrophy OR prostatic enlargement OR Urinary Bladder Neck Obstruction OR Prostate Adenoma OR benign prostatic hyperplasia OR bladder outlet obstruction OR MeSH descriptor Prostatic Hyperplasia explode all trees OR MeSH descriptor bladder outlet obstruction explode all trees OR MeSH descriptor Urinary Bladder Neck Obstruction explode all trees OR MeSH descriptor Prostate Adenoma explode all trees) AND (enucleation OR MeSH descriptor enucleation explode all trees) AND (open prostatectomy OR transvesical prostatectomy OR transvesical open prostatectomy OR transvesical open enucleation OR [retropubic](http://www.ncbi.nlm.nih.gov/pubmed/24729021) prostatectomy OR retropubic adenomectomy OR adenomectomy OR prostatectomy OR prostatectom* OR MeSH descriptor prostatectomy explode all trees)

(3) SCI-EXPANDED

(TS=(prostatic hyperplasia OR prostate hypertrophy OR prostatic enlargement OR Urinary Bladder Neck Obstruction OR Prostate Adenoma OR benign prostatic hyperplasia OR bladder outlet obstruction) AND TS=(enucleation) AND TS=(open prostatectomy OR transvesical prostatectomy OR transvesical open prostatectomy OR transvesical open enucleation OR [retropubic](http://www.ncbi.nlm.nih.gov/pubmed/24729021) prostatectomy OR retropubic adenomectomy OR adenomectomy OR prostatectomy OR prostatectom*)) AND (TS=((clinical OR control* OR placebo OR random*) NEAR (trial* OR group* OR study OR studies OR placebo OR controlled)) NOT TS=animal*)

(4) EMBASE:

((prostatic AND ('hyperplasia'/exp OR hyperplasia)) OR ('prostate'/exp OR prostate AND ('hypertrophy'/exp OR hypertrophy)) OR (prostatic AND enlargement) OR (urinary AND ('bladder'/exp OR bladder) AND ('neck'/exp OR neck) AND ('obstruction'/exp OR obstruction)) OR ('prostate'/exp OR prostate AND ('adenoma'/exp OR adenoma)) OR (benign AND prostatic AND ('hyperplasia'/exp OR hyperplasia)) OR ('bladder'/exp OR bladder AND outlet AND ('obstruction'/exp OR obstruction))) AND ('enucleation'/exp OR enucleation) AND (open AND ('prostatectomy'/exp OR prostatectomy) OR (transvesical AND ('prostatectomy'/exp OR prostatectomy)) OR (transvesical AND open AND ('prostatectomy'/exp OR prostatectomy)) OR (transvesical AND open AND ('enucleation'/exp OR enucleation)) OR (retropubic AND ('prostatectomy'/exp OR prostatectomy)) OR (retropubic AND adenomectomy) OR (adenomectomy) OR ('prostatectomy'/exp OR prostatectomy) OR (prostatectom*)) AND ((random* OR factorial* OR crossover* OR placebo*) OR ('crossover-procedure'/exp OR 'crossover-procedure' OR 'double-blind procedure'/exp OR 'double-blind procedure' OR 'randomised controlled trial'/exp OR 'randomised controlled trial' OR 'single-blind procedure'/exp OR 'single-blind procedure'))
