# Supplementary material for: Endoscopic Enucleation versus Open Prostatectomy for Treating Large Benign Prostatic Hyperplasia: A Meta-Analysis of Randomized Controlled Trials
Source: PLoS One. 2015 Mar 31;10(3):e0121265. doi: 10.1371/journal.pone.0121265 (PMC4380430; doi:10.1371/journal.pone.0121265)
Supplement: S2 File — Here's the DOIs necessary to access my data in the Table 3, Table 4 and Table 5. (DOCX) [file pone.0121265.s029.docx]

Here's the DOIs necessary to access my data in the table 3, table 4 and table 5.

1. Forest plot for operative time. <http://dx.doi.org/10.6084/m9.figshare.1287707>
2. Forest plot for catheterization. <http://dx.doi.org/10.6084/m9.figshare.1287706>
3. Forest plot for hospital stay. <http://dx.doi.org/10.6084/m9.figshare.1287709>
4. Forest plot for resected prostate weight. <http://dx.doi.org/10.6084/m9.figshare.1287711>
5. Forest plot for hemoglobin decrease. <http://dx.doi.org/10.6084/m9.figshare.1287708>
6. Forest plot for IPSS at 3-month. <http://dx.doi.org/10.6084/m9.figshare.1287715>
7. Forest plot for IPSS at 6-month. <http://dx.doi.org/10.6084/m9.figshare.1287716>
8. Forest plot for IPSS at 12-month. <http://dx.doi.org/10.6084/m9.figshare.1287717>
9. Forest plot for Qmax at 3-month. <http://dx.doi.org/10.6084/m9.figshare.1287724>
10. Forest plot for Qmax at 6-month. <http://dx.doi.org/10.6084/m9.figshare.1287725>
11. Forest plot for Qmax at 12-month. <http://dx.doi.org/10.6084/m9.figshare.1287726>
12. Forest plot for QoL at 3-month. <http://dx.doi.org/10.6084/m9.figshare.1287727>
13. Forest plot for QoL at 6-month. <http://dx.doi.org/10.6084/m9.figshare.1287728>
14. Forest plot for QoL at 12-month. <http://dx.doi.org/10.6084/m9.figshare.1287730>
15. Forest plot for PVR at 3-month. <http://dx.doi.org/10.6084/m9.figshare.1287720>
16. Forest plot for PVR at 6-month. <http://dx.doi.org/10.6084/m9.figshare.1287722>
17. Forest plot for PVR at 12-month. <http://dx.doi.org/10.6084/m9.figshare.1287723>
18. Forest plot for IIEF-5 at 3-month. <http://dx.doi.org/10.6084/m9.figshare.1287710>
19. Forest plot for IIEF-5 at 6-month. <http://dx.doi.org/10.6084/m9.figshare.1287712>
20. Forest plot for IIEF-5 at 12-month. <http://dx.doi.org/10.6084/m9.figshare.1287713>
21. Forest plot for IIEF-5 at 24-month. <http://dx.doi.org/10.6084/m9.figshare.1287714>
22. Forest plot for blood transfusion. <http://dx.doi.org/10.6084/m9.figshare.1287729>
23. Forest plot for recatheterization. <http://dx.doi.org/10.6084/m9.figshare.1287732>
24. Forest plot for urinary tract infection. <http://dx.doi.org/10.6084/m9.figshare.1287735>
25. Forest plot for urinary incontinence. <http://dx.doi.org/10.6084/m9.figshare.1287734>
26. Forest plot for BNC/urethral strictures. <http://dx.doi.org/10.6084/m9.figshare.1287731>
27. Forest plot for reintervention. <http://dx.doi.org/10.6084/m9.figshare.1287733>
